# Motivational Interviewing vs Case Management for Elder Abuse Intervention: A Comparison Study

**DOI:** 10.1093/geroni/igaf122.226

**Published:** 2025-12-31

**Authors:** Elsie Yan, Jasmin He, Daniel W L Lai, David Burnes, Karl Pillemer, Mark Lachs

**Affiliations:** The Hong Kong Polytechnic University, Hong Kong, Hong Kong, China (People’s Republic); The Hong Kong Polytechnic University, Hong Kong, Hong Kong, China (People’s Republic); Hong Kong Baptist University, Hong Kong, China (People’s Republic); University of Toronto, Toronto, Ontario, Canada; Cornell University, Ithaca, New York, United States; Weill Cornell Medicine, New York, New York, United States

## Abstract

Elder abuse is a serious public health issue. Considerable efforts have been made to develop interventions to address this issue. This study compared the effectiveness of a 4-session motivational interviewing (MI) program and an 8-session case management (CM) program in reducing abuse severity, promoting physical and mental health, general self-efficacy, social support, and motivation to change. Baseline data was collected from 129 older adults screening positive for elder abuse in a community survey. Assessments were repeated at the end of the intervention for the 86 cases receiving MI intervention and 43 cases receiving case management, and at 3-month follow up for 20 cases in the MI group. No demographic difference was observed between cases assigned to MI and CM at baseline. Wilcoxon signed-rank tests were conducted to examine intervention outcomes by different time points. At post-test, the MI group demonstrated significant improvement in abuse severity, psychological distress, general self-efficacy, social support and motivation to change (p<.001). The CM group also showed improvement in these areas with the exception of general self-efficacy (p<.01). Significant difference was observed between MI and CM group in their improvement in general self-efficacy. Abuse severity and psychological distress continued to drop at 3-month post-intervention (p<.05). Abuse severity and psychological distress continued to decrease between post-intervention and 3 month-follow-up (p<.05). Our findings suggest that MI is a cost-effective intervention for reducing abuse severity and risk.

